# Rational Therapeutics and Therapeuticians

**Published:** 1894-05-19

**Authors:** 


### The Hospital

An Institutional Journal of

The Medical Sciences and Hospital Administration, with

AN EXTRA NURSING SUPPLEMENT.

Vol. XVI.—No. 399.] [May 19, 1894.

### Rational Therapeutics and Therapeuticians.

Dr. Pye Smith tells us doctors that we are all rational in these modern times. At least, he told the fellows of the Hunterian Society that they all were; and as that society fairly represents all classes in the profession, we infer that he believes us all to be rational. But what is it to be rational? Merely to give up superstitious practices, or practices which are founded on abstract reasoning rather than upon observed facts and experiences, is not to establish a claim to rationality, reasonableness. There is such a thing as routine, such a thing as what is called "rule of thumb." Routine medical practice is very common, and though it may by accident now and again be rational, yet it is quite certain that it is often as irrational as irrational can be. To be rational is to be reasonable, and to be reasonable is to be mentally perfect. Who of us will affirm that even doctors are perfect?

It is very necessary for all persons and classes of persons who have a definite purpose in life to frequently take account of where they stand, or of the precise direction in which they are moving. Because if they happen to be moving in a wrong direction it is imperative that they should retrace their steps, and the sooner the better. The experience of medicine is somewhat striking in this regard. Not seldom has the medical profession moved in a given direction by almost unanimous consent of its members, and with a rapidity which might almost be called a "rush"; and not seldom has that rush been in an absolutely mistaken direction: and repentance and reformation have had to take the place of jubilance and self confidence. Let us remember the case of Koch and his tuberculin! What has happened before may certainly happen again. He would be a fool who would say otherwise.

What is that quality or method in modern medicine which justifies the claim of Dr. Pye Smith to call it "rational"? Is there in our art at the present moment such a distinctive quality or such a commanding method? We think there is; but we cannot agree with Dr. Pye Smith that the quality is so universally diffused, or the method so everywhere common in practice as to justify the high claim that the medical profession, taken as a whole, is a "rational" profession. It is only by degrading the word "rational" to a very low level of significance indeed that any such claim as this can be logically maintained. "Rule of thumb," we hold, is extensively prevalent, and we challenge the most ardent admirer of modernism to prove that rule of thumb, carried out by persons who are indifferent to the claims of any other rule, is one whit better than superstitious usages carried out by men who believed in their superstitions, and ardently desired the successful recovery of their patients. Superstition is by no means always the worst thing in the world, as we are sure Dr. Pye Smith will admit. There is a worse thing, and that is conscienceless idleness. Of conscienceless idleness in the medical, as in other professions, there is a plentiful supply.

The principles which animate and direct modern medicine are to a large extent rational. We take nothing for granted! That of itself is a noble principle, and absolutely essential to scientific progress. We use our eyes—we look, and look, and look again. That is surely the way to see, and seeing to know, as the Greeks held. We make experiments. This is the most distinguishing of all the marks of the modern medical art. We make experiments. We try if the therapeutic ship will sail, if the balloon will float in the air. Of all methods this, at any rate in principle, is the most definitely progressive and the most convincing. If a method will work, if it will give a satisfactory result, if it will do this once, twice, a hundred, ten thousand times, then we know that a law has been discovered or established, and that in medicine, as in other things, "that which has been is that which shall be." These are the rational principles upon which modern medicine is at least attempting to get itself built up. They are principles applied in their fulness in daily practice by one in a score, if even by so many. So long as it is the one in the twenty, and not the nineteen, who apply them, it is hardly consistent with elementary truth to call medicine a rational profession. As well might we call some of the Churches rational religions because one leading spirit in ten thousand exercises his reason and dares to give his rational conclusions voice.

Medicine is rational in that she professes to have forsaken all other lights for the light of pure reason. But what is medicine? Is it one great medical luminary here and there whose commanding intellect sees all things from the elevation of a kind of inner Eiffel Tower? That is not medicine; it is one man: one man who, whatever his calling, would still be a man of vision world-wide, and of conscience to do what he should see and know. It is easy to single out such a man, because there are not a few such in medicine, and by an act of imagination to transfer his personality to the whole profession. But it is not honest, and it is not profitable. The great world sees medicine as it is, and seeing, is by no means ready to put upon it the noble mint stamp of high intellectual capacity and sweet reasonableness.

Rational therapeutics can only be developed and established by means of rational persons. Rational persons are the most difficult of all persons to make. You can make partisans, you can make ambitious men, you can make routine medical practitioners, and routine men of all sorts without any difficulty whatever, by the thousand. But to make a man rational, to make him such that he is not only rational today, but will be rational to-morrow, rational next week, rational in every day and act of his life until death comes or second childhood takes possession of him—that is a task beyond the power of Dr. Pye Smith and the Hunterian Society. It is nevertheless the task of tasks which modern medicine has to accomplish. We all pray for the millennium of rational therapeutics: in order that that millennium may speedily dawn we should set ourselves to the conversion of every member of the medical profession into a rational therapeutician.

It is not, perhaps, desirable for us doctors to sound our trumpet of rationality too loudly. We are, after all, only a product of our age. The spirit of the age, the tremendous energy of competition, drives everybody in the direction of rationality. It is only as we see and do that which is rational that we do that which is extensively and assuredly successful. The irrational man may be successful, but he is successful only occasionally and by luck. The truly rational man is always successful where success is possible, and his success in medicine can be counted upon beforehand as certainly as in any other region of experience where the cause is the parent of the effect. Moreover, let us remember that, as yet at any rate, the province of our rationality is very limited; and not only is our rationality not wide, it is not yet very deep. We have recovered our rationalistic sight so recently that the best of us, in many regions of pathology and therapeutics, still "see men as trees walking." Many of us, as we have said, have not practically recovered our rational sight at all. Not superstition, but routine is the modern danger of the medical profession; and until routine gives place universally to reason the profession can make no general boast of its "rational therapeutics." If it is necessary for us to continually "clear our minds of cant," it is equally necessary for us constantly to clear them of self-delusion. As yet the idea of a rational therapeutic is a dream—a beautiful dream we grant. That the dream may one day become a reality we do not doubt. But it will be when the average man has laid aside more of the stubbornness of the savage and the selfishness of the wolf, and when the average doctor has strangled his prejudices and strengthened his courage so that he dares to think for himself and to carry his convictions into act and deed.

